# Adverse fetal and perinatal outcomes associated with Zika virus infection during pregnancy: an individual participant data meta-analysis

**DOI:** 10.1016/j.eclinm.2025.103231

**Published:** 2025-05-08

**Authors:** The Zika Virus Individual Participant Data Consortium, Edna Acosta Pérez, Juan Aguilar Ticona, Jackeline Alger, Celia Alpuche Aranda, Angélica Amado Niño, Emily Ansusinha, Thalia Araújo, Juan Arias, Lumumba Arriaga Nieto, Marcos Ávila, Azucena Bardají, Carlos Becerra Mojica, Andrea Benedetti, Ana Bertozzi, Sarah Bethencourt, Karen Blackmon, Victor Borja Aburto, Patrícia Brasil, Elizabeth Brickley, William Britt, Nathalie Broutet, Pierre Buekens, André Cabié, David Alejandro Cabrera-Gaytán, Rodrigo Cachay, Maria Luisa Cafferata, Isaac Caicedo-Castro, Juan Calle, Guilherme Calvet, Harlan Campbell, Maribel Campos, Mabel Carabali, Helen Cerigo, Celia Cordon, Juliana Silva Corrêa, Federico Costa, Conrado Coutinho, Antonio Cunha, Carlos Cure Cure, Johanna Damen, Marcela Daza, Ilich De la Hoz Siegler, Roberta DeBiasi, Thomas Debray, Valentijn de Jong, Camille Delgado-López, Geraldo Duarte, Gabriella Duarte Miranda, Valorie Eckert, Sophie Eickmann, Esther Ellis, Cassia Estofolete, Lester Figueroa Bolaños, Olivier Fléchelles, Kirsten Fong, Maria Barbara Franco Gomes, Trevon Fuller, Victoria Fumadó, Anna Funk, Luis Galvão, Gabriela Lopes Gama, Patrick Gérardin, Luz Gibbons, Anna Goncé, Amy Gonzalez, Eduardo Gotuzzo, Concepción Grajales-Muñiz, Paul Gustafson, Tahani Hamdan, Eva Harris, Cosme Harrison, Najeh Hcini, Cristina Hofer, Natanaël Holband, Claudia Hormiga Sánchez, Ivonne Huerta, Isabel Hurtado, Irene Inwani, David Cruvinel Isaac, Thomas Jaenisch, Esaú João, Amadu Juliana, Jose Paulo Pereira Junior, Edna Kara, Caron Kim, Albert Ko, Nancy Krebs, Angelle Desiree LaBeaud, Heather Lake-Burger, Jill Lebov, Yee-Sin Leo, Brooke Levis, Talita Lima, Simon Ling, Eduardo Lopez-Medina, Cynthia Lorenzo, Calum Macpherson, Olivia Manders, Elena Marbán-Castro, Ernesto Torres de Azevedo Marques, Celina Turchi Martelli, Flor Martinez-Espinosa, Gloria Martinó González, Gustavo Matta, Salim Máttar, Lauren Maxwell, John McCracken, Adriana Melo, Clara Menéndez, Marcela Mercado Reyes, María Consuelo Miranda Montoya, Demócrito de Barros Miranda-Filho, Ulisses Montarroyos, Karel Moons, Maria Elisabeth Moreira, Jack Moye, Sarah Mulkey, José Muñoz-Medina, Johanna Munoz, Marisa Mussi-Pinhata, Silvia Negrini, Nivison Nery, Jurg Niederbacher Velásquez, Karin Nielsen, M. Kariuki Njenga, Trevor Noël, Mauricio Nogueira, Theresa Ochoa, Consuelo Oliveira, Eric Osoro, Ester Paiva Souto, Miguel Parra-Saavedra, Saulo Passos, Luiza Pela Rosado, Bernadete Perez Coêlho, Priscila Cardia Petra, Léo Pomar, Arnaldo Prata-Barbosa, Ingrid Rabe, Mitermayer G. Reis, Ana Maria Rivera Casas, Diana Rojas, Kerstin Daniela Rosenberger, Paola Mariela Saba Villarroel, Nuria Sanchez Clemente, Magda Sanz Cortes, Janet Sayers, Deolinda Scalabrin, Lavinia Schuler-Faccini, Stacey Schultz-Cherry, Aluisio Segurado, Kirstin Short, Priya Shreedhar, Antônio Silva, Ronaldo Silva, Vivian Avelino Silva, Isadora Cristina de Siqueira, Karen Sohan, Antoni Soriano-Arandes, Carmen Soria-Segarra, Paulo Sucasas da Costa, Adriana Tami, Jousilene Tavares, Maria Benamor Teixeira, Robert F. Terry, Tun-Linn Thein, Soe Soe Thwin, Frank Tobian, Vivian Torres Rodríguez, Isabel Trejos, Marilia Dalva Turchi, César Ugarte-Gil, Miguel Valencia-Prado, Alfonso Vallejos-Parás, Zilton Vasconselos, Ana Beatriz Gorini da Veiga, Carmen Viñuela-Benéitez, Manon Vouga, Yinghui Wei, Jamie Westcott, Marc-Alain Widdowson, Ricardo Ximenes, Carmen Zorrilla

**Keywords:** Zika virus, Pregnancy, Congenital Zika Syndrome, Individual participant data meta-analysis, Microcephaly, Perinatal outcomes

## Abstract

**Background:**

Zika virus (ZIKV) infection during pregnancy is associated with an increased risk of congenital malformations. The prevalence of short and long-term consequences, however, remains uncertain due to heterogeneity across studies. Individual Participant Data Meta-Analysis (IPD-MA) offers an alternative approach to provide more precise and generalisable estimates through data harmonisation across studies, allowing for standardised definitions and exploration of heterogeneity. This project was undertaken to estimate absolute and relative risks of adverse outcomes for individuals with ZIKV infection during pregnancy.

**Methods:**

IPD-MA studies and their datasets were identified through a systematic search conducted in 2018 with the following criteria: observational longitudinal or surveillance-based studies investigating ZIKV during pregnancy or at birth, measured fetal, infant, or child outcomes, and included at least 10 participants. Here we used IPD data shared by March 2022 from 18 studies from international health organisations and research networks, comprising 24 unique datasets, in 11 countries. Datasets were harmonised with standardised definitions, using variables related to pregnant individuals, methods used for ZIKV diagnoses, fetal characteristics and outcomes, and pooled for analysis. Frequentist and Bayesian regression methods were applied to estimate outcome prevalence and evaluate the association between maternal ZIKV infection and fetal loss, microcephaly and congenital zika syndrome as primary outcomes.

**Findings:**

Data including 9568 pregnant individuals and 9608 newborns, were harmonised. The risk of severe primary microcephaly was significantly higher in ZIKV-positive pregnancies (1.5%, CI 0.8%–2.7%) compared to ZIKV-negative ones (0.3%, CI 0.1%–1.0%), with a relative risk of 4.5 (CI 1.5–13.3) in the one-stage meta-analysis. While some risk estimates were consistent between Bayesian and Frequentist methods, estimates for other outcomes varied, underscoring the influence of both the analytical approach and the definition of ZIKV on the associations.

**Interpretation:**

Our findings align with previously published meta-analyses and indicate an added burden to adverse pregnancy outcomes with higher prevalence compared to pre-epidemic population-based average values. Future research should focus on additional outcomes with clear definitions of maternal infection. Women of reproductive age should be informed about the risks of Zika infection during pregnancy to support reproductive planning.

**Funding:**

This project was supported by the 10.13039/100010269Wellcome Trust grant number 206532/Z/17/Z, the 10.13039/100004423WHOHealth Emergencies Programme Global Arbovirus Initiative, and the 10.13039/100004423WHO Department of Sexual and Reproductive Health and Research, including the Human Reproduction Special Programme (HRP).

**Research in context**

**Evidence before this study**

Previous studies on adverse outcomes of Zika virus (ZIKV) infection during pregnancy have been limited by small sample sizes, variations in definitions, and differences in methods. While systematic reviews and meta-analyses of aggregated data have been conducted, there has yet to be a large-scale individual participant data meta-analysis (IPD-MA) that thoroughly evaluates both the absolute and relative risks across multiple countries using standard methodologies. Studies eligible to participate in the IPD-MA were identified through a systematic search registered on PROSPERO (CRD42017068915). Datasets were identified via searches of Medline and Embase on 8 July 2018, without language restrictions, supplemented by expert consultations and monthly PubMed alerts. Eligible studies were longitudinal, observational, or surveillance-based, with ZIKV testing during pregnancy at birth, measured fetal, infant, or child outcomes, and included at least 10 participants.

**Added value of this study**

This study presents an IPD-MA that integrates data from 18 studies conducted across 11 countries, involving 9568 pregnant women and 9608 newborns. By standardising definitions and applying both frequentist and Bayesian analytical methods, we provide a robust assessment of the absolute and relative risks of microcephaly, fetal loss, and CZS in pregnancies affected by ZIKV compared to those that are not infected. Additionally, this study examines the effects of various analytical methodologies and identifies potential sources of heterogeneity.

**Implications of all the available evidence**

Our findings contribute to a more refined understanding of the risks associated with ZIKV infection during pregnancy, informing public health policies, clinical guidelines, and preparedness efforts for future outbreaks. The results underscore the need for continued surveillance, early diagnosis, and targeted interventions to mitigate adverse outcomes associated with CZS.

## Introduction

Zika virus (ZIKV) is mostly transmitted by infected *Aedes* mosquitoes, though sexual transmission through unprotected intercourse and blood transfusion transmission have been described.^1^ ZIKV infection during pregnancy is associated with an increased risk of congenital malformations, including microcephaly and neurological manifestations. Congenital Zika Syndrome (CZS) is defined as a specific collection of congenital malformations and disorders caused by prenatal exposure to ZIKV.^2^^,^^3^ Without approved vaccines or specific treatments, clinical management focuses on symptom mitigation and providing care to ZIKV-infected pregnancies and their offspring.^4^, ^5^, ^6^

A systematic review estimated the prevalence of microcephaly among infants born from ZIKV-infected mothers at 3% (95% confidence interval [CI] 2%–5%) and a risk of fetal loss of 4% (CI 2%–6%).^7^ Prevalence estimates of other malformations ranged from <1% (e.g., ventriculomegaly) to 6% for central nervous system malformations.^8^^,^^9^ However, the prevalence of short and long-term consequences remains uncertain due to heterogeneity across studies, including differences in ZIKV infection ascertainment, outcome definitions, and follow-up durations.^8^ Differences in gestational age at the time of ZIKV infection across studies may also contribute to differences in the outcome's frequency. Furthermore, most studies have small sample sizes, are regionally limited to Latin America, and describe a limited set of outcomes.^9^^,^^10^ Traditional meta-analyses relying on aggregated data with varying definitions are at risk of classification and evaluation bias in the definitions of ZIKV infection and outcomes.^11^, ^12^, ^13^, ^14^

Individual Participant Data Meta-Analysis (IPD-MA) offers an alternative approach, analysing individual-level data for more precise and generalisable estimates.^15^ IPD-MA includes data harmonisation across studies, allowing for standardised definitions and exploration of heterogeneity.^16^ Three consortia were created aiming to conduct IPD-MA to overcome the limitations of individual studies. They present different scales and territorial coverage. The Zika Brazilian Cohorts Consortium groups 15 cohorts in Brazil, followed in all regions of the country in which the epidemic occurred.^9^^,^^15^ The European Commission-supported consortia (ZIKAlliance, ZIKAction, and ZikaPLAN) has a larger number of studies, including data from 17 centers in 7 Latin American and Caribbean countries,^17^ but these studies are less homogeneous. The World Health Organization (WHO) Consortium is the more comprehensive, gathering the data from 54 participating sites from 22 participating countries and territories, but presents more heterogeneity across studies. These consortia are complementary to provide further insight into the consequences of ZIKV infection during pregnancy.

In 2016 and 2017, the Pan American Health Organization/World Health Organization (PAHO/WHO) supported meetings to harmonise ZIKV research protocols,^18^ leading to the establishment of the ZIKV Individual Participant Data (ZIKV-IPD) Consortium in 2017.^10^^,^^19^^,^^20^ This consortium, involving multiple international health organisations and research networks leading studies of pregnant individuals with Zika virus, aimed to develop and validate prognostic models for predicting adverse fetal and perinatal outcomes related to ZIKV infection during pregnancy, to guide healthcare practice. These models would favor the implementation of prenatal ZIKV screening programs, enhancing established antenatal care by providing evidence for decision–making while weighing the benefits and potential harms of screening.^21^

We present here the first steps to reaching these goals. The objective of this IPD-MA was to estimate the absolute and relative risks of microcephaly, CZS and fetal loss for women who did and did not experience ZIKV infection during pregnancy.

## Methods

### Search strategy and selection criteria

The study protocol for the IPD-MA, the systematic review (PROSPERO CRD42017068915), the ZIKV-IPD Consortium metadata survey, and the search strategy were previously described.^10^^,^^19^ Briefly, datasets were identified via systematic searches of Medline and Embase including ZIKV (e.g., ‘Zika virus’ or ‘Zika fever’) and maternal and pregnancy related terms (e.g., pregnan∗ or matern∗ or gestation∗ or perinatal∗ or birth∗ or congenital∗ or newborn∗ or fetal or fetus∗ or foetal or foetus∗ or neonat∗ or infan∗ or toddler∗ or child∗) on 8 July 2018, date identified via consortium consultation after establishment of the consortium in 2017. There were no language restrictions, and the search was supplemented by expert consultations, contacting health ministries and authorities such as the WHO, and by monthly PubMed alerts. Eligible studies were observational longitudinal, or surveillance-based studies (e.g., healthcare surveillance-based studies in which participant's enrolment is based on knowledge of the exposure status i.e., including ZIKV-positive only cases and potentially their outcomes), with ZIKV testing during pregnancy or at birth, which measured fetal, infant, or child outcomes, and included at least 10 participants. Ineligible studies were narrative reviews, studies without ZIKV testing during or case series with less than 10 individuals.^19^ Principal investigators (PI) of eligible studies, identified by the review (i.e., open to all researchers and not restricted to consortium members), were invited to complete a metadata survey and to share data. The metadata survey contained questions related to ZIKV testing, outcome definitions, and covariate definitions and measurements. Briefly, the duration of enrolment for ZIKV-IPD participating studies in the metadata survey ranged from 1 to 60 months (median = 18; Q1–Q3: 11–28 months), and the median duration of the follow-up was 24 months (Q1–Q3 15–29 months) with a maximum of 60 months. Other results are presented elsewhere.^10^

### Selection bias

**Fig. 1:**
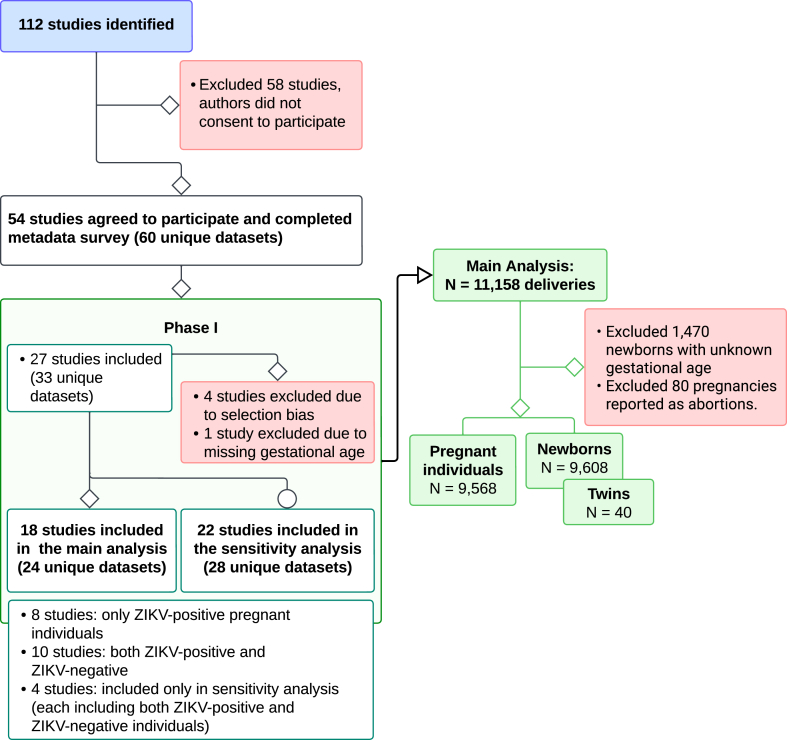
Study selection.

In this phase, we assessed IPD participating studies for selection bias, particularly regarding whether knowledge of an infant's condition—such as microcephaly, CZS, or miscarriage-might have influenced participation. Each study was classified as low, moderate, or high risk for selection bias using a four-question questionnaire developed independently from the metadata survey. The questions were: (1) *Could a woman's decision to participate in the study have been impacted by her knowing about the microcephaly status of her infant?* (2) *Could a woman's decision to participate in the study have been impacted by her knowing about the CZS status of her infant?* (3) *Could a woman's decision to participate in the study have been impacted by her knowing something about the miscarriage status of her infant?* (4) *Did the possible status of the infants (microcephaly, CZS, miscarriage) influence the recruitment of women in the study?* Hence, this phase included studies with confirmed ZIKV cases (e.g., eight ZIKV-positive cohorts) and a mix of ZIKV-positive and -negative participants, with low risk of selection bias (Fig. 1, Table S1). Those potentially biased by the participation of women with affected children were included only in the sensitivity analysis.

### Data harmonisation

Eligible studies that shared data underwent harmonisation by expert teams focusing on ZIKV exposure, covariates, and outcomes. This process involved aligning study-specific definitions and data collection methods with our developed standardised definitions, ensuring consistency across datasets. The harmonisation process included reviewing the raw data, mapping variables from each study to the WHO set of 234 key variables and resolving discrepancies in definitions or measurements. Through this process, the teams harmonised 80 variables related to the pregnant individual (demographics, socioeconomic status, medical and pregnancy history, current medical condition, pregnancy complications and intrauterine exposures), 27 on ZIKV symptoms experienced by the pregnant individual, 50 on ZIKV diagnostics performed on samples from the pregnant individual, five on ZIKV diagnostics for the fetus, 39 on fetal characteristics and outcomes, 32 on infant and child ZIKV-diagnostics, and one on infant and child death and autopsy.

### Outcomes

The primary outcomes considered were miscarriage at <20 weeks gestation, fetal loss at ≥20 weeks gestation, microcephaly regardless of diagnosis timing, and CZS. Secondary outcomes included early fetal death (20–27 weeks gestation), late fetal death (≥28 weeks gestation), primary microcephaly, gestational age at birth, birth weight, neurologic congenital abnormalities, and non-neurologic congenital abnormalities. Due to limited data (five recorded miscarriages), the risk of miscarriage was not estimated. Per the WHO interim guidelines, primary microcephaly was defined as head circumference at birth more than 2 standard deviations (SD) below the average for sex and gestational age, per INTERGROWTH-21st standards, expecting about 2.28% prevalence or 228 per 10,000 in a typical population.^8^^,^^22^ For gestational age beyond 42 weeks, the estimate for 42 weeks was used. Severe microcephaly was defined as head circumference at birth more than 3 SDs below the average for sex and gestational age, per INTERGROWTH-21st standards, expecting about 0.13% prevalence or 13 per 10,000 in a typical population. In cases where insufficient data was available to determine microcephaly status, microcephaly status as reported by the study was used. CZS was defined as confirmed maternal or fetal ZIKV infection with either severe microcephaly at birth or other malformations (e.g., limb contractures, high muscle tone, eye abnormalities, and hearing loss) as per WHO.^4^ Congenital Zika was identified by at least one of these manifestations in children without microcephaly born to women with laboratory evidence of ZIKV during pregnancy. See Supplementary Material, Table S3, for a comprehensive overview of variables.

### Statistics

Study population characteristics, including demographics, socioeconomic status, medical and pregnancy history, current medical conditions, pregnancy complications, and intrauterine exposures, are described according to the distribution of each variable.

Maternal exposure to ZIKV was defined using the study-specific definition (sZIKV), which was confirmed by the PI, and a standardised definition (stdZIKV), which categorizes evidence of infection as robust, moderate, limited, or no evidence of infection following the algorithm by Ximenes et al.^23^ Robust, moderate, and limited evidence were collapsed as ZIKV-positive and no evidence as ZIKV-negative. See Supplementary Material, Table S2.

We considered frequentist and Bayesian approaches to estimate outcome prevalence and evaluate the association between maternal ZIKV infection and study outcomes. One-stage meta-analysis (where data from all studies are pooled first and study-specific random intercept adjusted for heterogeneity), and two-stage meta-analysis (where parameter estimation was done for each study separately and then pooled), were conducted on datasets where missing data were imputed using multilevel multiple imputation methods. While one-stage IPD-MA uses data from all studies to estimate absolute risk and relative risk, two-stage meta-analysis could be conducted only for studies where both ZIKV positive and negative groups were enrolled. Similar to one stage meta-analysis, Bayesian analysis used the full dataset of all studies and reduced uncertainty from missingness by including in the model four variables related to ZIKV exposure status. Gaussian priors, centered on expected outcome event rate, were considered, and study-specific random intercept models were fitted to estimate absolute and relative risks.

For the frequentist analysis, we used multiple imputations with chained equations (MICE) to generate 50 imputed datasets. We used one-stage and two-stage IPD-MA approaches applying random-effects mixed binomial models with a log link to estimate the relative risk (RR), and logit link with back transformation to estimate the absolute risk (AR) separately in ZIKV positive and in ZIKV negative women, with corresponding 95% CIs.^16^ For the frequentist analyses, the RR was estimated exclusively from studies that included both ZIKV-positive and ZIKV-negative individuals to ensure valid comparisons. For Bayesian analysis, a random-effects logistic regression model with logit link function and a hierarchical prior specification was fit to the full data (i.e., ZIKV-positive and in ZIKV-negative women together) to obtain the posterior estimates of the ARs and the RR, with 95% credible intervals (CrI).^24^ The target population is a hypothetical population from which each of the study populations are sampled and neither frequentist nor Bayesian models incorporated any covariate adjustment. Heterogeneity across studies was addressed through study selection, definitions harmonisation, and statistical modeling, as described previously. Individual study and pooled estimates are presented in forest plots to visually assess variability. Details of imputation, frequentist and Bayesian analyses are described in the Supplementary Material, Table S4.

### Ethics

The project, along with comprehensive documentation, was submitted to the World Health Organization Ethics Review Committee. Since the study aimed to analyse previously collected de-identified data, it was deemed exempt from review.

### The role of funding source

Funding from the Wellcome Trust grant (number 206532/Z/17/Z) and the WHO Health Emergencies (WHE) Programme Global Arbovirus Initiative to the WHO Department of Sexual and Reproductive Health and Research - Human Reproduction Programme (HRP), enabled HRP to convene the WHO ZIKA IPD-MA consortium. The consortium was responsible for designing the study, developing the protocol and coordinating expert groups for data harmonisation, analysis, interpretation, and writing of the report. Funders did not have any role in study design, data collection, data analysis, interpretation and report writing. Two technical experts from the WHE Programme contributed to the harmonisation of exposure variables and reviewed the manuscript. HRP contributed to establishing the web-based data collection platform for management of data collection.

The authors alone are responsible for the views expressed in this publication and such views do not necessarily represent the views, decisions, or policies of the institutions with which they are affiliated.

## Results

Of 112 eligible studies, PIs from 54 studies agreed to participate in the consortium and completed the metadata survey. Some studies had multiple sites and contributed multiple datasets, while the design, ZIKV assays, and outcome ascertainment were the same. Out of the 54 studies, 27 studies (33 datasets) were shared before March 2022 and were considered in this phase of the analysis. Following the bias assessment, four studies were excluded for selection bias; one study was excluded for lack of gestational age data, and four studies were considered for sensitivity analysis only. Hence, the main analysis comprised 18 studies with 24 unique datasets. The sensitivity analysis comprised 22 studies with 28 unique datasets (Fig. 1, Table S1).

There was considerable agreement between study-specific and standardised definitions of maternal ZIKV infection with Prevalence- and Bias-Adjusted Kappa >0.5 for 14 of 22 studies (Table S5). There was a wide range in prevalence of neonatal outcomes reported, with 5/22 studies observing over 50% of newborns with microcephaly, 5/22 studies observing over 5% with CZS, and 4/22 studies reporting >3% fetal loss in their study population (Table S6).

Focusing on the 18 studies included in the primary analysis, six were conducted in Brazil, and eight of these studies recruited only ZIKV-positive individuals. After exclusion for unconfirmed gestational age (1470 of 11,158 pregnancies) and early termination of pregnancy by abortion (80 records), the final dataset for primary analysis included 9568 pregnant individuals and 9608 newborns, accounting for 40 multiple births (Fig. 1). Study-specific sample sizes ranged between 46 and 4058 newborns.

The median age of pregnant individuals was 27 years (Q1–Q3: 22–32), and the median gestational age at sZIKV infection was 20 weeks (Q1–Q3: 13–28). In total, per the study definition, 5928 (62.0%) newborns were born to sZIKV-positive and 2348 (24.5%) to sZIKV-negative pregnant individuals, and 1292 (13.5%) had missing information. Fetal ZIKV status was available for 678 (7.1%) newborns only, of whom 145 (21.4%) were ZIKV-positive and born to sZIKV-positive pregnant individuals. Among 77.1% of newborns with information on microcephaly at birth, 681 of 7410 (9.2%) had primary microcephaly per study definition. CZS status, per the study definition, was available for 6316 (65.7%) newborns, with 146 (2.3%) positive diagnoses. Of those, 139 (95.2%) were born to sZIKV-positive pregnant individuals (Table 1). Other malformations included neurological abnormalities (77 cases), contractures (one case), gastrointestinal (two cases), ocular (two cases), non-neurological (two cases), and congenital abnormalities excluding primary microcephaly (24 cases). Fetal loss status was reported in all 9380 pregnancies at ≥20 weeks, with 40 losses (1.5%) among sZIKV-negative and 14 (0.2%) among sZIKV-positive pregnancies.

**Table 1:** Maternal and newborn characteristics by Zika infection status as defined by each contributing study for the 18 studies in the main analysis.

Characteristic	ZIKV study definition (N, %)^a^			Overall (N, %) (N = 9568)	Unadjusted p-value^b^
	Negative (N = 2817)	Positive (N = 6391)	Unclassified^c^ (N = 360)		
**Age (years)**					
Mean (SD)	27.6 (6.2)	26.7 (6.1)	23.7 (6.5)	27.1 (6.3)	0.0001
Median [Q1, Q3]	27.0 [23.0, 32.0]	27.0 [22.0, 31.0]	22.0 [18.0, 28.0]	27.0 [22.0, 32.0]	
Missing	131	4961	45	5137 (54.7)	
**Education**					
No education	6 (0.3)	131 (14.7)	1	138 (1.4)	<0.0001
Primary school	541 (26.2)	60 (6.7)	79	680 (7.1)	
Secondary school	863 (41.8)	234 (26.3)	89	1186 (12.4)	
Incomplete tertiary education	219 (10.6)	403 (45.3)	89	711 (7.4)	
Bachelor's degree	408 (19.8)	45 (5.1)	9	462 (4.8)	
Graduate or professional degree	27 (1.3)	17 (1.9)	5	49 (0.5)	
Missing	753	5501	88	6342 (66.3)	
**Body mass index (used Pre-pregnancy weight, in kg)**					
Mean (SD)	24.9 (4.0)	26.8 (6.5)	18.8 (NA)	26.8 (6.4)	0.0071
Median [Q1, Q3]	24.3 [22.0, 28.0]	25.7 [21.9, 30.9]	18.8 [18.8, 18.8]	25.6 [21.9, 30.8]	
Missing	2716	3685	361	6762 (70.7)	
**Gestational age at end of pregnancy**					
Mean (SD)	38.6 (4.0)	37.6 (4.8)	37.9 (5.6)	37.9 (4.7)	<0.0001
Median [Min, Max]	39.0 [38.0, 40.0]	39.0 [37.2, 39.1]	39.0 [38.0, 40.0]	39.0 [38.0, 40.0]	
**Trimester at end of pregnancy**					
First: 6–13 weeks	23 (0.8)	97 (1.5)	8	128 (1.3)	<0.0001
Second: 14–27 weeks	29 (1.0)	60 (1.0)	8	97 (1.0)	
Third: 28–40 weeks	2145 (76,7)	5719 (90.9)	268	8132 (85.0)	
Post-term: 41 weeks and beyond	600 (21.5)	415 (6.6)	75	1090 (11.4)	
Missing	20	100	1	121 (1.3)	
**Maternal zika infection by Ximenes et al. definition**					
Negative	2147 (99.2)	167 (2.8)	34 (89.5)	2348 (28.4)	–
Limited	0 (0)	0 (0)	0 (0)	0 (0)	
Moderate	15 (0.7)	3863 (63.6)	4 (10.5)	3882 (46.9)	
Robust	2 (0.1)	2044 (33.7)	0 (0)	2046 (24.7)	
Missing	653	316	322	1292	
**Newborns**					
Number of newborns (row %)	2845 (29.6)	6401 (66.6)	362 (3.8)	9608	–
Fetal ZIKV infection yes	0 (0)	145 (36.5)	0	145 (15.1)	<0.0001
No	215 (100)	252 (63.5)	66	533 (5.6)	
Missing	2630	6004	296	8930 (92.9)	
Microcephaly yes	167 (16.4)	505 (8.3)	9	681 (7.1)	<0.0001
No	854 (83.6)	5552 (91.7)	323	6729 (70.0)	
Missing	1824	344	30	2198 (22.9)	
CZS yes	7 (1.5)	139 (2.5)	0	146 (1.5)	0.1628
No	473 (98.5)	5398 (97.5)	299	6170 (64.2)	
Missing	2365	864	63	3292 (34.3)	
Congenital Zika yes	35 (12.9)	559 (15.9)	4	598 (6.2)	0.2308
No	237 (87.1)	2947 (84.1)	38	3222 (33.5)	
Missing	2573	2895	320	5788 (60.2)	

a Study sample sizes may differ from those in the original dataset, due to exclusions based on the criteria outlined in this manuscript.

b For categorical variables, Pearson's Chi-squared test or Fisher's Exact test is used. For continuous variables, the Wilcoxon rank sum test is used to compare medians.

c The unclassified group is not considered when calculating p-values.

Arbovirus-related symptoms during the current pregnancy were reported in 41.8% of the pooled study population, with rash being the most predominant symptom reported (Table 2). Table S7 summarizes the birth statistics of newborns based on the standardised ZIKV definition.

**Table 2:** Maternal symptoms by Zika infection status as defined by each contributing study for the 18 studies in the main analysis.

Characteristic	ZIKV study definition (N, %)^a^			Overall (N, %) (N = 9568)	Unadjusted p-value^b^
	Negative (N = 2446)	Positive (N = 6294)	Unclassified (N = 828)		
**Any arbovirus-related symptoms during the current pregnancy**					
Yes	851 (30.3)	3131 (51.3)	21	4003 (41.8)	<0.0001
No	1960 (69.7)	2974 (48.7)	295	5229 (54.7)	
Missing	6	286	44	336 (3.5)	
**Fever**					
Yes	271 (10.6)	1214 (35.1)	14	1499 (15.7)	<0.0001
No	2293 (89.4)	2247 (64.9)	302	4842 (50.6)	
Missing	253	2930	44	3227 (33.7)	
**Rash**					
Yes	200 (7.5)	2736 (78.9)	14	2950 (30.8)	<0.0001
No	2450 (92.5)	732 (21.1)	302	3484 (36.4)	
Missing	167	2923	44	3134 (32.8)	
**Muscle Pain**					
Yes	75 (14.4)	549 (35.6)	11	635 (6.6)	<0.0001
No	444 (85.6)	994 (64.4)	31	1469 (15.4)	
Missing	2298	4848	318	7464 (78.0)	
**Arthralgia**					
Yes	454 (17.4)	849 (29.6)	12	1315 (13.6)	<0.0001
No	2148 (82.6)	2016 (70.4)	304	4468 (46.7)	
Missing	215	3526	44	3785 (39.6)	
**Headache**					
Yes	481 (26.6)	832 (25.3)	4	1317 (13.8)	0.3100
No	1327 (73.4)	2456 (74.7)	0	3783 (39.5)	
Missing	1009	3103	356	4468 (46.7)	
**Bleeding**					
Yes	23 (1.4)	4 (0.5)	0	27 (0.3)	0.0396
No	1599 (98.6)	809 (99.5)	0	2408 (25.2)	
Missing	1195	5578	360	7133 (74.6)	
**Sore throat**					
Yes	80 (4.3)	46 (6.4)	0	126 (1.3)	0.0274
No	1769 (95.7)	670 (93.6)	38	2477 (25.9)	
Missing	968	5675	322	6965 (72.8)	

a Study sample sizes may differ from those in the original dataset, due to exclusions based on the criteria outlined in this manuscript.

b Pearson's Chi-squared, or Fisher's Exact test is used.

**Fig. 2:**
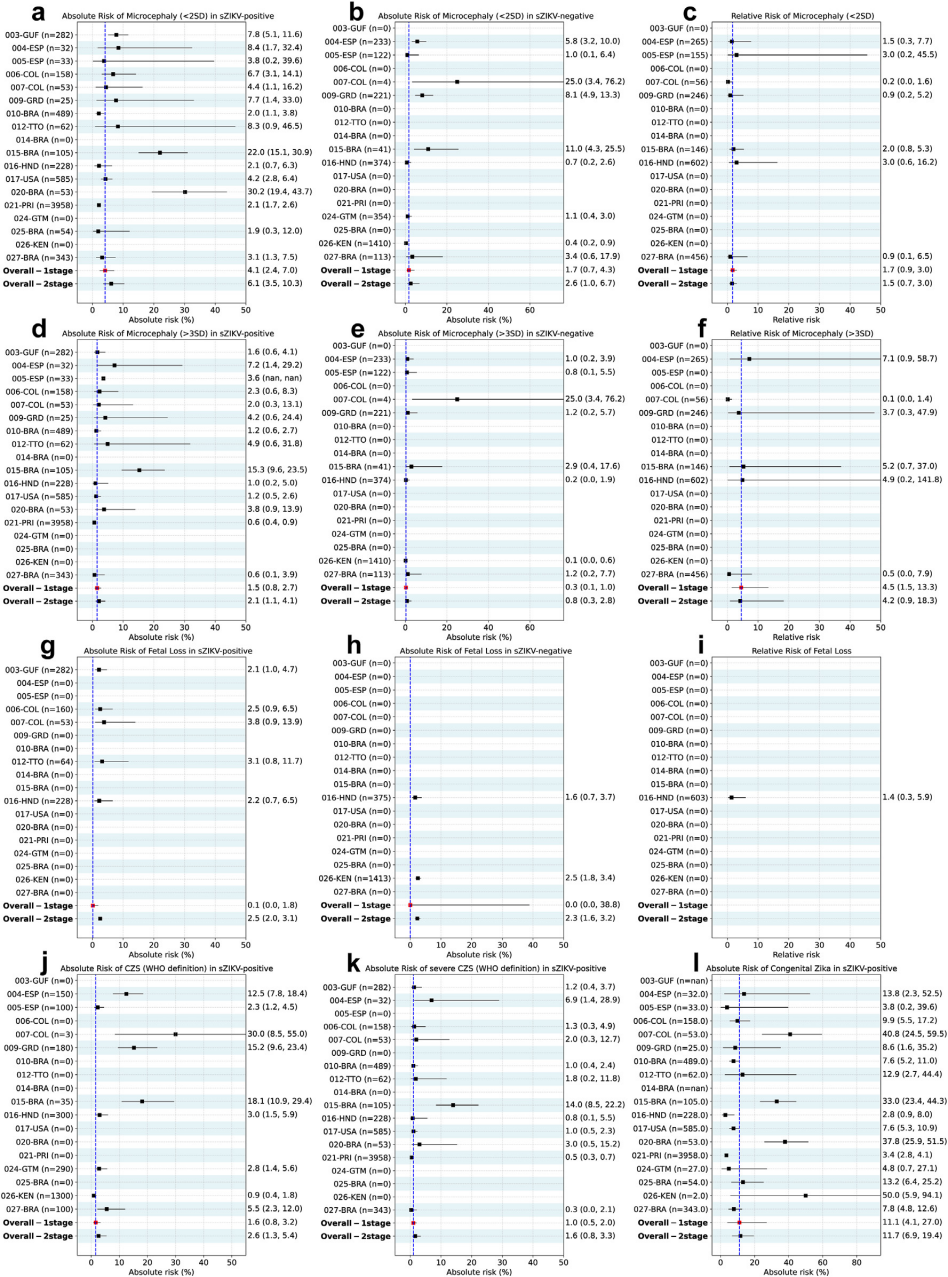
(a) Absolute Risk of Microcephaly (<2SD) in sZIKV-positive, (b) Absolute Risk of Microcephaly (<2SD) in sZIKV-negative, (c) Relative Risk of Microcephaly (<2SD), (d) Absolute Risk of Microcephaly (>3SD) in sZIKV-positive, (e) Absolute Risk of Microcephaly (>3SD) in sZIKV-negative, (f) Relative Risk of Microcephaly (>3SD), (g) Absolute Risk of Fetal Loss in sZIKV-positive, (h) Absolute Risk of Fetal Loss in sZIKV-negative, (i) Relative Risk of Fetal Loss, (j) Absolute Risk of CZS (WHO definition) in sZIKV-positive, (k) Absolute Risk of severe CZS (WHO definition) in sZIKV-positive, (l) Absolute Risk of Congenital Zika in sZIKV-positive. Absolute and relative risks of microcephaly, fetal loss, and Congenital Zika Syndrome (CZS), stratified by maternal ZIKV infection status. Error bars indicate 95% confidence intervals (CI). Squares represent study-specific estimates and diamonds the overall pooled estimates (red: one-stage meta-analysis; blue: two-stage meta-analysis). Dashed vertical lines indicate no-effect values. Abbreviations: BRA, Brazil; COL, Colombia; ESP, Spain; GUF, French Guiana; GRD, Grenada; GUA, Guadeloupe; MTQ, Martinique; REU, Réunion; VEN, Venezuela; ZIKV, Zika virus; CZS, Congenital Zika Syndrome; WHO, World Health Organization; SD, Standard deviations.

In the frequentist analysis, the AR of primary microcephaly (2SD) for newborns of sZIKV-positive women was 4.1% (CI 2.4%–7.0%) and 1.7% (CI 0.7%–4.3%) for newborns of sZIKV-negative individuals, with a RR of 1.7 (CI 0.9–3.0) in the one-stage meta-analysis, indicating no statistically significant difference in risk between sZIKV-positive and sZIKV-negative pregnancies. In the Bayesian analysis, the posterior median estimate for the risk of primary microcephaly in newborns of sZIKV-positive pregnant individuals was 3.9% (CrI 1.5%–8.8%). For sZIKV-negative individuals, the posterior estimate for the AR was 2.0% (CrI 0.8%–4.1%). The posterior RR was 2.0 (CrI 0.9–4.3), measuring the association between maternal sZIKV infection and microcephaly (Table 3, Fig. 2a–c).

**Table 3:** Absolute and relative risk with 95% confidence intervals for pregnancy outcomes by ZIKV infection status estimated by Frequentist and Bayesian analytic approaches.

Outcome	Estimate^a^	Frequentist				Bayesian	
		with study specific definition of ZIKV		with standardised definition of ZIKV		with study specific definition of ZIKV	with standardised definition of ZIKV
		One-stage	Two-stage	One-stage	Two-stage		
Microcephaly at birth (2SD)	Absolute risk ZIKV+ (%)	4.1 [2.4, 7.0]	6.1 [3.5, 10.3]	3.7 [2.3, 6.1]	5.2 [3.0, 8.7]	3.9 [1.5, 8.8]	2.5 [0.7, 7.1]
	Absolute risk ZIKV− (%)	1.7 [0.7, 4.3]	2.6 [1.0, 6.7]	3.7 [1.5, 9.2]	7.6 [2.5, 20.6]	2.0 [0.8, 4.1]	3.1 [1.3, 7.4]
	Relative risk	1.7 [0.9, 3.0]	1.5 [0.7, 3.0]	0.6 [0.3,1.1]	0.4 [0.2, 1.0]	2.0 [0.9, 4.3]	0.8 [0.3, 1.9]
Microcephaly at birth (3SD)	Absolute risk ZIKV+ (%)	1.5 [0.8, 2.7]	2.1 [1.1, 4.1]	1.2 [0.6, 2.4]	1.9 [0.9, 3.6]	1.5 [ 0.6, 3.2]	0.8 [0.1, 2.9]
	Absolute risk ZIKV− (%)	0.3 [0.1, 1.0]	0.8 [0.3, 2.8]	0.7 [0.2, 2.8]	3.2 [0.7, 13.8]	0.3 [0.1, 0.6]	0.4 [0.1, 1.1]
	Relative risk	4.5 [1.5, 13.3]	4.2 [0.9, 18.3]	0.6 [0.3, 1.1]	0.4 [0.2, 1.0]	6.2 [2.2, 18.0]	2.0 [0.4, 7.6]
Fetal loss	Absolute risk ZIKV+ (%)	0.1 [0.0, 1.8]	2.5 [2.0, 3.1]	0.1 [0.0, 1.8]	2.2 [1.6, 2.9]	0.1 [0.01, 0.7]	0.1 [0.01, 0.7]
	Absolute risk ZIKV− (%)	0.01 [0.0, 38.8]	2.3 [1.6, 3.2]	0.01 [0.0, 16.8]	3.1 [1.1, 8.7]	0.5 [0.1, 2.1]	0.5 [0.1, 2.0]
				0.83 [0.48, 2.00]	0.27 [0.04, 2.00]	0.2 [0.02, 1.6]	0.21 [0.02, 1.24]
CZS (microcephaly 2SD)	Absolute risk ZIKV+ (%)	1.6 [0.8, 3.2]	2.6 [1.3, 5.4]	1.2 [0.6, 2.7]	2.3 [1.1, 4.7]	2.0 [0.9, 4.6]	2.3 [0.9, 6.2]
CZS (severe (3SD) microcephaly	Absolute risk ZIKV+ (%)	1.0 [0.5, 2.0]	1.6 [0.8, 3.3]	0.8 [0.4, 1.7]	1.4 [0.7, 2.8]	1.6 [0.7, 3.5]	1.8 [0.7, 5.0]
Congenital Zika	Absolute risk ZIKV+ (%)	11.1 [4.1, 27.0]	11.7 [6.9, 19.4]	7.9 [4.4, 13.6]	15.4 [5.9, 34.5]	23.0 [5.5, 56.8]	20.3 [4.7, 53.3]

a For the frequentist analysis the 95% confidence interval based on the imputed dataset is provided while for the Bayesian analysis the 95% credible intervals based on the raw dataset.

AR of severe primary microcephaly (3SD) was 1.5% (CI 0.8%–2.7%) for newborns of sZIKV-positive pregnant individuals and 0.3% (CI 0.1%–1.0%) for newborns from sZIKV-negative pregnancies, with RR of 4.5 (CI 1.5–13.3) in the one-stage meta-analysis, indicating statistically significant difference in risk. In the Bayesian analysis, the posterior median estimate for the risk of severe microcephaly in newborns of sZIKV-positive individuals was 1.5% (CrI 0.6%–3.2%). For sZIKV-negative individuals, the posterior median estimate was 0.3% (CrI 0.1%–0.6%). The posterior median estimate for the RR was 6.2 (CrI 2.2–18.0) (Table 3, Fig. 2d–f).

AR of fetal loss in sZIKV-positive pregnant individuals ranged between 2.1% and 3.8% across studies, with an overall AR of 0.1% (CI 0.0%–1.8%) for sZIKV-positive and 0.01% (CI 0.0%–38.8%) for sZIKV-negatives. In Bayesian analysis, the posterior median estimate for the AR of fetal loss in sZIKV-positive pregnant individuals was 0.1% (CrI 0.01%–0.7%). For sZIKV-negative pregnancies, the posterior estimate was 0.5% (CrI 0.1%–2.1%). The posterior estimate for the RR was 0.2 (CrI 0.02–1.6) (Table 3, Fig. 2g–i).

In the frequentist analysis, the AR of CZS using microcephaly (2SD) in its definition for sZIKV-positive women ranged between 0.9% and 30.0%, with an overall AR of 1.6% (CI 0.8%–3.2%) in the one-stage meta-analysis. Using the severe primary microcephaly (3SD) in the definition, the AR in sZIKV-positive individuals was 1.0% (CI 0.5%–2.0%). In Bayesian analysis, the posterior estimate for the AR of CZS in newborns of sZIKV-positive pregnant individuals was 2.0% (CrI 0.9%–4.6%). Using the severe primary microcephaly in the definition of the CZS, the AR in sZIKV-positive individuals was 1.6% (CI 0.7%–3.5%). In the frequentist analysis, in the one-stage meta-analysis, the AR for congenital Zika was 11.1% (CI 4.1%–27.0%) in sZIKV-positive individuals. In the Bayesian analysis, the posterior estimate for the AR was 23.0% (Crl 5.5%–56.8%), (Table 3, Fig. 2j–l).

### Sensitivity analyses

After including four additional studies in the sensitivity analyses to assess the impact of exclusions and selection bias, the results remained consistent with the primary findings. Detailed sensitivity analysis results are provided in the Supplementary Material (Table S8).

## Discussion

In Phase-I of this large IPD-MA, we assessed the absolute and relative risks of fetal and infant outcomes in women with and without ZIKV infection during pregnancy. We found that the pooled absolute risk of microcephaly at birth for newborns born of ZIKV-infected individuals ranged from 2.5% to 6.1%, depending on the analytical approach used, consistent with previous meta-analyses reporting risks below 6%. Among ZIKV-positive pregnancies, Bayesian analyses generally estimate a lower risk of microcephaly compared to the Frequentist analyses, particularly under the standardised definition. For fetal loss, the absolute risk ranged from 0.1% to 2.5% for ZIKV-positive pregnancies, similar to other studies reporting risks between 0% and 11%.^8^^,^^9^^,^^25^^,^^26^ The risk of fetal loss did not differ significantly between ZIKV–infected and non-infected pregnancies as per study definitions, but these estimates were subject to substantial uncertainty.

Previous meta-analyses on ZIKV related outcomes include Martins et al.,^8^ who reported a prevalence of congenital microcephaly of 3% (CI 2%–5%) based on 16 studies; Nithiyanantham and Badaw (2017),^25^ reported a prevalence of 3.9% (CI 2.4%–5.4%) based on 21 studies; and Coelho and Crovella (2017)^26^ reported a prevalence of 2.3% (CI 1.0%–5.3%) based on eight studies. Our findings align with these estimates and are consistent with Ximenes et al., based on an IPD-MA of 1548 women with RT-PCR-confirmed ZIKV infection during pregnancy, reported an absolute risk of primary microcephaly at 2.6% (CI 1.1%–4.5%) at the first evaluation and 4% (CI 2.0%–6.6%) during follow-up.^9^

These prevalences are notably higher than pre-epidemic general population values, such as the Latin American Collaborative Study of Congenital Malformations (ECLAMC), which, using the data derived from 107 hospitals in 10 South American countries, reported a microcephaly prevalence of 3.0 (CI 2.7–3.4) per 10,000 births from 2005 to 2014. This estimate was based on microcephaly as defined by individual pediatricians' diagnoses, referencing ECLAMC's standard of defining microcephaly as 3 standard deviations below the mean. Similarly, the European Surveillance of Congenital Anomalies (EUROCAT), covering 15 countries, reported 1.5 (CI 1.2–2.0) per 10,000 births from 2003 to 2012, using data from 24 EUROCAT registries. Definitions varied across registries, incorporating both the 2SD and 3SD thresholds and individual clinical criteria.^27^^,^^28^

In comparing the study-specific and standardised ZIKV infection definitions, we found a higher prevalence of primary microcephaly among infants born to ZIKV-negative individuals using the standardised definition. The variation in prevalence was wider among ZIKV-negative individuals (1.7%–7.6%) compared to ZIKV-positive individuals (2.5%–6.1%). This discrepancy is likely due to misclassification, as non-systematic testing throughout pregnancy may have led to some exposed individuals being classified as non-exposed. Also, the short time window for PCR positivity and the lack of ZIKV seroconversion data among pregnant individuals, as previously reported,^15^ may have contributed to false negative results. Additionally, 50% of the studies did not enroll ZIKV-negative individuals according to their own ZIKV definitions, possibly skewing our ZIKV-negative sample's representativeness.

The relative risk for primary microcephaly was higher in ZIKV–infected pregnancies using the study-specific definition for the reasons already described. Although results were not statistically significant, the point estimates suggest an increased risk, particularly for severe primary microcephaly, indicative of more severe brain damage characteristic of CZS. No meta-analyses to date have included ZIKV-negative individuals, and case–control studies have shown a stronger association between ZIKV infection and microcephaly.^29^, ^30^, ^31^ This is expected as in these studies the association is based on ZIKV laboratory evidence in children, while in cohort studies it is based on laboratory evidence in pregnant women. Note that not all children born to infected women are infected in utero, and not all infected fetuses present symptoms.^32^

Although unable to explore the type of fetal losses, our findings align with Ximenes et al., who reported a miscarriage risk of 0.9%, and a stillbirth risk of 0.3%,^9^ which are lower than the estimated fetal loss of 4.0% (CI 2.0%–6.0%) reported by Martins et al., which included both miscarriage and stillbirth.^8^ These results should be interpreted cautiously due to varying gestational ages at enrolment across studies, affecting the pregnancy denominator and risk estimates, particularly for fetal loss earlier in pregnancy. No prior meta-analysis provided pooled estimate of relative risk for fetal loss, and we did not observe a higher risk in ZIKV-positive pregnancies.

Our study has strengths and limitations. This IPD-MA is large and geographically diverse, including studies from North, Central, and South America, Africa, and Europe and examines absolute and relative risks of adverse fetal and perinatal outcomes related to ZIKV infection during pregnancy. It includes data from 18 studies across 11 countries worldwide, comprising 6391 ZIKV-positive and 2817 ZIKV-negative individuals. While one study contributes a substantial proportion of the sample size, the inclusion of multiple studies from diverse regions helps mitigate concerns about population representativeness. This large sample size enhances statistical power but introduces challenges due to heterogeneity in study designs, settings, and definitions.

To mitigate these challenges, we employed one-stage and two-stage frequentist analyses and Bayesian analyses to estimate absolute and relative risks. Prevalence estimates were higher in the two-stage analysis, likely because it first calculates study-specific estimates, which can amplify heterogeneity before pooling. Due to substantial missing data and the small number of microcephaly cases, different analytical methods—each handling missingness differently—yielded varying estimates. Additionally, heterogeneity stemmed from varied study durations, follow-up periods, and differing gestational ages at enrolment.

Variability in ZIKV-infection ascertainment methods and outcome definitions also contributed to heterogeneity. Despite applying standardised definitions, some variation persisted due to differences in laboratory techniques and test results. Misclassification of outcomes, particularly primary microcephaly, may have occurred due to head circumference measurement inaccuracies.^33^

Due to data sparsity, our analysis could not explore miscarriage, and early miscarriages may not have been captured. While we acknowledge the importance of adjusting for confounders, including gestational age or trimester, our results are the first from such a large dataset, with further adjustments planned in Phase II of our analysis.

Imputing missing data involves decisions that influence risk estimates, described as a ‘garden of forking paths'. To address this, two independent teams used distinct statistical approaches, and we reported both sets of results, enhancing transparency and reproducibility.

Though primary data from all studies was not retrospective, the information to assess studies for selection bias was obtained from the PIs retrospectively, and we cannot exclude that some degree of information bias may have occurred.

To ensure transparency and reproducibility, we conducted sensitivity analyses to assess the impact of exclusions and potential selection bias, and the results are presented in the Supplementary Material.

This IPD-MA has advanced in relation to previous studies by grouping individual data from several studies in different regions of the world where the Zika epidemic occurred and providing robust estimates of the absolute and relative risks of adverse outcomes from ZIKV infection during pregnancy. However, some points need to be further explored. Future research should focus on additional outcomes with standardised definitions of maternal infection, while appropriately addressing heterogeneity. Phase-II of this analysis will incorporate more datasets, explore additional outcomes, enhance understanding of the exposure, and identify factors that modify the risk of adverse events for pregnant individuals and children.

Our findings can support the planning of actions aimed at the care to be provided to mothers who may become infected during pregnancy and to their children. Women of reproductive age should be informed about the risks of Zika infection during pregnancy to support reproductive planning. Pregnant individuals should be offered testing and informed about potential risks to make informed decisions. Psychological support should be available, and children born to ZIKV-infected pregnant individuals should receive comprehensive evaluations for early diagnosis and management of congenital Zika manifestations.

## Contributors

The coordinating team (Broutet N, Carabali M, Jaenisch T, Kara E, Kim C, Maxwell L, Sayers J, Silva R, Thwin SS, and Ximenes R) conceptualized the study design and coordinated its data collection and management.

The exposure working group (Alger J, Alvares DR, Brasil P, Calvet G, Cerigo H, Cunha A LaBeaud D, Marques E, Martelli CT, Mattar S, Passos SD, Rabe I, Scalabrin D, and Veiga ABG), defined exposure variables for data harmonisation.

The outcome working group (Araújo TVB, Arrieta G, Avelino-Silva V, Bardají A, Bertozzi A, Blackmon K, Buekens P, Cachay R, Clemente NS, da Costa PS, de Siqueira I, DeBiasi RL, Duarte G, Eickmann S, Fumadó V, Gerardin P, Hofer C, Holband N, Lee E, Lopez-Medina E, Miranda-Filho DB, Mojica CB, Moreira ME, Mulkey SB, Mussi-Pinhata M, Noel T, Pomar L, Prata-Barbosa A, Sohan K, Soria-Segarra C, Soriano-Arandes A, Ticona JPA, Valencia D, Viñuela-Benéitez C, and Vouga M), defined outcome variables for data harmonisation.

The analytic working group (Benedetti A, Caicedo-Castro I, Campbell H, Damen JAAG, Debray T, de Jong V, Gibbons L, Gustafson P, Hofer C, Montarroyos U, Moons K, Munoz J, and Wei Y) conducted statistical analysis, synthesized results, and interpreted them.

The data harmonisation and metadata survey group (Cerigo H, De La Hoz-Siegler I, Levis B, Rosenberger KD, Shreedhar P, and Tobian F), worked on data harmonisation and metadata survey.

The social science working group (Acosta E, Campos M, da Silva B, Daza M, Gomez A, Gama G, Hormiga C, Manders O, Marban-Castro E, Matta G, Melo A, Mercado M, Miranda MC, Paiva E, Petra P, Pimentel C, Torres V, and Vega V) conducted qualitative research on the social impact of Zika.

The writing group (Carabali M, Campbell H, Kara E, Kim C, Silva R, Thwin SS, and Ximenes R) drafted and revised the manuscript, ensuring an accurate representation of the study.

Campbell H, Damen JAAG, Munoz J, and Silva S directly accessed and verified the underlying data reported in the manuscript. All members of the Zika Virus Individual Participant Data Consortium read and approved the final version of the manuscript.

The complete list of the WHO ZIKV IPD-MA Consortium members is provided in the Supplementary File ZIK IPD-MA CONSORTIUM_list of names.docx.

## Data sharing statement

Individual datasets contributed to the “The Zika Virus Individual Participant Data Consortium” are the property of the individual contributors and will not be shared in Open access format. Harmonised and pooled analytic dataset with anonymised data will be available for request upon completion of both Phase I and Phase II data acquisition, harmonisation, analysis, and publication. Following the WHO research data share policy and with agreement from the consortium members, aggregated data by study will be available for download from an easily accessible webpage on a WHO Portal via a designated link. The requestor may register their contact information and submit the reason for the request and planned use. A data editorial board will meet at regular intervals to review requests. Data will be provided in both proprietary and non-proprietary and open-standard formats by using widely accepted formats for data files.

## Declaration of interests

We declare no competing interests.

## Appendix A Supplementary data

Supplementary Tables

Collaborative authors
