# Supplementary material for: Adverse fetal and perinatal outcomes associated with Zika virus infection during pregnancy: an individual participant data meta-analysis
Source: eClinicalMedicine. 2025 May 8;83:103231. doi: 10.1016/j.eclinm.2025.103231 (PMC12235389; doi:10.1016/j.eclinm.2025.103231)
Supplement: Supplementary Tables [file mmc1.docx]

**Adverse fetal and perinatal outcomes associated with Zika virus infection during pregnancy: an individual participant data meta-analysis**

The Zika Virus Individual Participant Data Consortium

**SUPPLEMENTARY MATERIAL**

**Contents**

[Table S1: Summary of all studies with datasets contributed for prior to March 2022 2](#_Toc193355447)

[Table S2: Mapping of study definition of ZIKV infection (sZIKV) to standardized maternal ZIKV (stdZIKV) infection status 3](#_Toc193355448)

[Table S3: Definition of Participant-level variables of interest 3](#_Toc193355449)

[Table S4. Description of analytic methods 4](#_Toc193355450)

[Table S5: Maternal ZIKV infection status and agreement between study definition and standardized definition 6](#_Toc193355451)

[Table S6: Pregnancy and neonatal outcomes by study 7](#_Toc193355452)

[Table S7: Newborns' birth statistics Based on the standardized ZIKV definition 7](#_Toc193355453)

[Table S8: Sensitivity analysis with 23 studies: absolute and relative risk estimates based on different statistical analysis 7](#_Toc193355454)

[REFERENCES 9](#_Toc193355455)

# Table S1: Summary of all studies with datasets contributed for prior to March 2022

| **Study ID & Country** | **DOI of most recent related publication** | **Participant Details** | **ZIKV Criteria & Ascertainment** | **Time frame** | **Potential risk of Selection Bias** |
| --- | --- | --- | --- | --- | --- |
| 001-BRA,  Brazil | - | Reproductive age; Knowledge about the CZS status of infant influenced participation | Symptom/Exposure; Molecular Diag. | Mar 2016-  Dec 2017 | 🔴 High |
| 002-BRA,  Brazil^Ø^ | https://doi.org/10.1371/journal.pone.0200168 | Pregnant; Community | Lab-confirmed; Molecular Diag., Immunoassay, RDT | Jan 2015-  Aug 2016 | 🟡 Moderate |
| 003-GUF,  French Guiana | https://doi.org/10.1136/bmj.k4431 | Pregnant; Hospital/Health Facility | Lab-confirmed; Molecular Diag. + Immunoassay | Jan 2016-  Jul 2016 | 🟢 Low |
| 004-ESP,  Spain | https://doi.org/10.3390/children9101537 | Pregnant; Travelers | Symptom/Exposure; Molecular Diag. + Immunoassay | Jun 2016-  Jun 2020 | 🟢 Low |
| 005-ESP,  Spain | https://doi.org/10.1016/j.tmaid.2021.101985 | Pregnant; Hospital/Health Facility | Lab-confirmed/Symptom; Clinical Case Def., Mol. Diag., Immuno. | Jan 2016-  Dec 2017 | 🟢 Low |
| 006-COL, Colombia | https://doi.org/10.1097/INF.0000000000002307 | Pregnant; Community | Lab-confirmed; Molecular Diag. | Jul 2016-  Dec 2017 | 🟢 Low |
| 007-COL, Colombia | https://doi.org/10.1001/jamapediatrics.2019.5204 | Pregnant; Community, Travelers | Symptom/Exposure; Clinical Case Def., Mol. Diag., Immuno., RDT | Jun 2016-  Jun 2017 | 🟢 Low |
| 008-USA,  USA | https://doi.org/10.1001/jamapediatrics.2019.5204 | Pregnant; Hospital/Facility | Symptom/Exposure; Clinical Case Def., Mol. Diag., Immuno. | May 2016-  Nov 2019 | 🟡 Moderate |
| 009-GRD, Grenada | https://doi.org/10.1136/archdischild-2020-321031 | Pregnant/Delivered; Hospital/Facility | Symptom/Exposure; Molecular Diag. + Immunoassay | Jan 2017-  Jan 2018 | 🟢 Low |
| 010-BRA,  Brazil | https://obgyn.onlinelibrary.wiley.com/doi/full/10.1111/1471-0528.16490 | Pregnant; Community, Hospital/Facility | Lab-confirmed; Molecular Diag. + Immunoassay | Jan-16 | 🟢 Low |
| 011-BRA,  Brazil | https://doi.org/10.1177/0883073820983163 | Pregnant; Hospital/Facility | Lab-confirmed/Symptom; Mol. Diag. + RDT | Jan 2016-  Sep 2020 | 🟡 Moderate |
| 012-TTO, Trinidad and Tobago | https://doi.org/10.1002/ijgo.12313 | Pregnant; Hospital/Health Facility | Lab-confirmed; Clinical Case Def. + Mol. Diag. | Mar 2016-  Sep 2016 | 🟢 Low |
| 013-BRA,  Brazil | https://doi.org/10.1038/s41598-020-69235-0 | Pregnant; Hospital/Facility | Unrelated to ZIKV; Molecular Diag. | Mar 2016-  Oct 2018 | 🟡 Moderate |
| 014-BRA,  Brazil | https://doi.org/10.1371/journal.pone.0256444 | Pregnant; Community | Unrelated to ZIKV; Immunoassay | Jan 2015-  May 2016 | 🟢 Low |
| 015-BRA,  Brazil | https://doi.org/10.1371/journal.pntd.0009612. | Delivered; Hospital/Health Facility | Unrelated to ZIKV; Immunoassay | Oct 2015-  Jan 2016 | 🟢 Low |
| 016-HND, Honduras | https://doi.org/10.4269/ajtmh.20-1483 | Pregnant; Hospital/Health Facility | Unrelated to ZIKV; Molecular Diag. | Jul 2016-  May 2021 | 🟢 Low |
| 017-USA,  USA | https://doi.org/10.15585/mmwr.mm7103a1 | Pregnant/Delivered; Community, Travelers | Lab-confirmed; Molecular Diag. + Immunoassay | Dec 2015-  Mar 2018 | 🟢 Low |
| 018-COL, Colombia | https://doi.org/10.3390/tropicalmed6040183 | Pregnant; Hospital/Facility | Symptom/Exposure; Clinical Case Def., Mol. Diag., Immuno. | Apr 2016-  Oct 2017 | 🟡 Moderate |
| 019-BRA,  Brazil | https://doi.org/10.1002/ijgo.13042 | Newborns with microcephaly or from mother with ZIKV | Symptom/Exposure; Clinical Case Def. | Feb 2016-  Dec 2016 | 🔴 High |
| 020-BRA,  Brazil | https://doi.org/10.1093/trstmh/trz098 | Delivered; Hospital/Health Facility | Symptom/Exposure; Clinical Case Def. + Immunoassay | Feb 2016-  Dec 2016 | 🟢 Low |
| 021-PRI,  Puerto Rico | https://doi.org/10.15585/mmwr.mm6731e1 | Pregnant/Delivered; Community, Hospital/Facility | Lab-confirmed; Molecular Diag. + Immunoassay | Jan 2015-  Mar 2018 | 🟢 Low |
| 022-BRA,  Brazil | https://doi.org/10.1093/tropej/fmad030. PMID: 37705261 | Pregnant; Hospital/Health Facility | Symptom/Exposure; Molecular Diag. | Feb 2016-  Aug 2017 | 🔴 High |
| 023-BRA,  Brazil | https://doi.org/10.1016/j.ijid.2021.02.072 | Infants with CZS; Hospital/Facility | Molecular Diag. | Jan 2016-  May 2019 | 🔴 High |
| 024-GTM, Guatemala | https://doi.org/10.1056/NEJMoa1604037 | Pregnant; Hospital/Health Facility, Travelers | Unrelated to ZIKV; Clinical Case Def., Mol. Diag., Immuno. | Dec 2016-  Oct 2017 | 🟢 Low |
| 025-BRA,  Brazil | https://doi.org/10.1016/j.cmi.2017.11.004 | Pregnant; Hospital/Health Facility | Lab-confirmed; Molecular Diag. | Feb 2016-  Jun 2016 | 🟢 Low |
| 026-KEN,  Kenya | https://doi.org/10.1186/s12916-022-02498-8 | Pregnant; Hospital/Health Facility | Symptom/Exposure; Clinical Case Def., Mol. Diag., Immuno. | Oct 2017-  Aug 2019 | 🟢 Low |
| 027-BRA,  Brazil | https://doi.org/10.1016/ S1473-3099(16)30318-8 | Pregnant; Community, Hospital/Facility | Lab-confirmed/Symptom; Molecular Diag. + Immunoassay | Feb 2016-  Aug 2020 | 🟢 Low |

Ø study qualified for the sensitivity analysis but was excluded due to all observations lacking gestational age data, based on the criteria outlined in this manuscript.

# Table S2: Mapping of study definition of ZIKV infection (sZIKV) to standardized maternal ZIKV (stdZIKV) infection status

| **Study definition of maternal ZIKV (sZIKV):**  pregnant woman diagnosed with ZIKV during this pregnancy using clinical, laboratory diagnosis or self-report as defined by the study | **Standardized definition of maternal ZIKV (stdZIKV):**  **Ximenes et al. Algorithm** |
| --- | --- |
| **Positive** | **Robust evidence:**  (RT-qPCR positive in pregnancy) **OR** (IgM seroconversion in pregnancy) **OR** (PRNT seroconversion in pregnancy) **OR** (IgM positive in pregnancy **AND** PRNT titer >= 20 in pregnancy) **OR** (IgM positive in pregnancy **AND** PRTN seroconversion within 6 months post-pregnancy) **OR** (IgM positive in pregnancy **AND** PRNT titer >= 20 within 6 months post-pregnancy) **OR** (IgG3 positive in pregnancy **AND** PRNT titer >= 20 in pregnancy) **OR** (IgG3 positive in pregnancy **AND** PRTN seroconversion within 6 months post-pregnancy) **OR** (IgG3 positive in pregnancy **AND** PRNT titer >= 20 within 6 months post-pregnancy) |
| **Positive** | **Moderate evidence:**  (IgM positive in pregnancy) **OR** (IgG3 positive in pregnancy) **OR** (PRNT titer >= 1000 in pregnancy AND rise in titer measured within 2 months post-pregnancy) **OR** (PRNT 4-fold rise from titer in pregnancy to titer within two months post-pregnancy) **OR** (RT-qPCR **AND** PRNT titer >= 100 within 3 months post-pregnancy) **OR** (IgM in pregnancy **AND** PRNT titer >= 100 within 3 months post-pregnancy) |
| **Positive** | **Limited evidence:**  (PRNT titer >= 100 in pregnancy) **OR** (PRNT titer >= 100 within 1-month post-pregnancy) **OR** (PRNT titer >= 100 within 2-3 months post-pregnancy) **OR** (PRNT titer >= 100 within 2-3 months post-pregnancy) **OR** (PRNT titer >= 100 within 2-3 months post-pregnancy **AND** IgM positive within 2-3 months post-pregnancy) **OR** (PRNT titer >= 100 within 4-6 months post-pregnancy) OR (PRNT titer >= 20 & < 100 in pregnancy) **OR** (non-negative PRNT titer in pregnancy) **OR** (PRNT titer >= 20 & < 100 within 1 month post-pregnancy) **OR** (non-negative PRNT titer within 1-month post-pregnancy) |
| **Negative** | **Negative evidence:**  (RT-qPCR negative in pregnancy **AND** IgM, IgG3 and PRNT negative in pregnancy) **OR** (RT-qPCR negative in pregnancy **AND** IgM and PRNT negative in pregnancy) **OR** (RT-qPCR negative in pregnancy **AND** IgM and IgG3 negative in pregnancy) **OR** (RT-qPCR negative in pregnancy **AND** IgM negative in pregnancy) **OR** (IgM negative in pregnancy **AND** RT-qPCR not tested in pregnancy **AND** IgG3 and PRNT negative in pregnancy) **OR** (IgM negative in pregnancy **AND** RT-qPCR not tested in pregnancy **AND** PRTN negative in pregnancy) **OR** (IgM negative in pregnancy **AND** RT-qPCR not tested in pregnancy **AND** PRNT and IgG3 not tested in pregnancy) |

# Table S3: Definition of Participant-level variables of interest

| **Group** | **Variable** | **Status** | **Definition** |
| --- | --- | --- | --- |
| **Exposure** | **Maternal ZIKV infection** | Available | Two variants:  1) Study definition  2) Following paper by Ximenes et al.^22^ |
|  | **Fetal or placental ZIKV infection** | High percentage of missing data | Documented laboratory confirmed ZIKV infection in placental or fetal tissues |
| **Primary outcomes** | **Miscarriage** | Available in some datasets | Loss of the product of the gestation occurred before 20 weeks of gestation. |
|  | **Fetal loss** | Available | Pregnancy loss (any loss of the product of the gestation at or after 20 weeks of gestation) |
|  | **Microcephaly** | Available | Three variants:  1) Study definition  2) Standardized definition (2 standard deviations below the mean)  3) Standardized definition (3 standard deviations below the mean) |
|  | **Congenital Zika Syndrome** | Available | Three variants:  1) Study definition  2) WHO definition– with microcephaly^4^  3) WHO definition – with severe microcephaly^4^ |
| **Secondary fetal outcomes** | **Induced abortion with microcephaly** | Not present in harmonized dataset | Voluntary or recommended termination of the gestation. |
|  | **Early fetal death** | Available | Fetal demise occurred between 20-27 weeks of gestation |
|  | **Late fetal death (≥28 weeks gestation)** | Available | Fetal demise occurred after 28 weeks of gestation |
|  | **Late fetal death (≥28 weeks gestation) with microcephaly** | Not present in harmonized dataset | Fetal demise occurred after 28 weeks of gestation and microcephaly was also present |
|  | **Placental insufficiency** | Not present in harmonized dataset | Documented placental insufficiency, presented as confirmed, probable, unlikely. |
|  | **Intrauterine growth restriction** | High percentage of missing data | Evidence of Intrauterine Growth Restriction (IGR), including fetal anthropometrics and weight |
| **Secondary infant outcomes** | **Gestational age at birth** | Available | Gestational age in weeks at time of delivery or c-section |
|  | **Birth weight** | Available | Weight in grams of the newborn |
|  | **Craniofacial abnormalities** | High percentage of missing data | Presence of any other cranium (head abnormalities other than microcephaly) combined with facial abnormalities |
|  | **Neuroimaging abnormalities** | Available | Identification by fetal or neonatal ultrasound or MRI of any of the described central nervous system abnormalities including but not restricted to: intracranial calcification, lissencephaly, hydranencephaly, porencephaly, ventriculomegaly, posterior fossa abnormalities, cerebellar hypoplasia, corpus callosal and vermian dysgenesis; focal cortical dysplasia |
|  | **Postnatal intraventricular hemorrhage** | Not present in harmonized dataset | Identification by MRI of intraventricular hemorrhage |
|  | **Motor abnormalities** | Not present in harmonized dataset | Abnormalities related to motor skills of the newborn, including but not restricted to hypotonia, hypertonia, hyperreflexia, spasticity, clonus, extrapyramidal symptoms |
|  | **Seizures, epilepsy** | Not present in harmonized dataset | Documented seizures at birth or perinatal period |
|  | **Ocular abnormalities (blindness, other)** | High percentage of missing data | Documented ocular abnormalities at birth or follow-up |
|  | **Congenital deafness or hearing loss** | High percentage of missing data | Documented hearing abnormalities at birth or follow-up |
| **Covariates** | **Arbovirus symptoms** | Available | Documented presence of any arbovirus-related symptoms during the current pregnancy, including fever, myalgia, arthralgia, cephalea, rash |
|  | **Preterm labor** | Available | Initiation of the labor towards delivery before 37 weeks of gestation: Very Early Pre-term (<28 weeks); Early Pre-term (28-31 weeks); Moderate Pre-term (32-33 weeks); Late Preterm (34-36 weeks) |
|  | **Confirmation of arboviral infection** | Available | Documented diagnosis of any arboviral disease during the current pregnancy: ZIKV, DENV, CHIKV, other |
|  | **Congenital contractures** | High percentage of missing data | arthrogryposis, uni or bilateral clubfoot |
|  | **Other non-neurologic congenital abnormalities** | Available | - |
| **Secondary outcomes detected after the infant period** | **Cortical auditory processing** | Not present in harmonized dataset | - |
|  | **Neurodevelopment** | Not present in harmonized dataset | Expressive and receptive language, fine and gross motor skills, attention and executive function, memory and learning, socioemotional development, overall neurodevelopmental score. |
|  | **Vision** | Not present in harmonized dataset | Cardiff test |

# Table S4. Description of analytic methods

| **Frequentist IPD Meta-analysis** | **Bayesian IPD Meta-analysis** |
| --- | --- |
| **Definition** | |
| Utilizes classical statistical methods to estimate parameters and test hypotheses. | Bayesian statistical methods address uncertainty in missing data and model parameters while incorporating prior knowledge. They are well-suited for estimating unknown quantities, such as the risk of microcephaly, from data with missingness. |
| **Handling of Imputation** | |
| We used multiple imputation with chained equations (mice) to handle missing values, in exposures and outcomes, generating 50 imputed datasets as per our protocol.^19^ A predictor matrix of 19 variables with the study-specific definition of maternal ZIKV infection serving as a critical predictor variable for imputation. | In the Bayesian analysis, a distinct approach was used to handle missing data by integrating four variables into the model that might serve as predictors of ZIKV status: (1) “arbovirus symptoms” - a binary variable indicating if the pregnant individual experienced any arbovirus-related symptoms (e.g., fever, myalgia, arthralgia, headache, rash, etc.) during the current pregnancy; (2) “Preterm labor” - a binary variable indicating if the pregnant individual experienced labor before 37 weeks of gestation for the current pregnancy; (3) “Birth weight of child” - birth weight in grams (<12 hours after delivery); and (4) “Confirmation of current arbovirus infection, indicating laboratory confirmation of dengue, chikungunya or other arboviral infection”.^8,16^  Since ZIKV infection status is likely a predictor for these four variables, knowledge of these variables may help reduce the uncertainty in parameter estimation due to missingness. We used the following priors for the parameters corresponding to these variables.  We defined Gaussian priors for intercept parameters with a mean of 0 and a standard deviation of the square root of 2. For parameters corresponding to the association between the auxiliary variables and ZIKV infection, we defined Gaussian priors with a mean of 0 and a standard deviation of square root of 10. We also included the study-specific definition of microcephaly status as a variable for which microcephaly status may be a predictor, and the study-specific definition of ZIKV as a variable for which ZIKV status may be a predictor. We assumed *a priori* that the study-specific definitions had reasonable predictive power with sensitivity and specificity ranging between 0.83 and 0.96 (with 50% prior probability). |
| **Imputation: predictor matrix and priors** | |
| The imputation predictor matrix included 19 variables:   1. Gestational Age 2. Sex of the fetus/infant 3. Head circumference at birth or up to 24 hours after birth (occipital-frontal at birth; ideally, average of three measurements), in cm 4. Microcephaly ever diagnosed, as defined by the study. Microcephaly could be diagnosed in prenatal, infant or child period. 5. Zika test result of the pregnant women with evidence according to Ximenes et al. (2019) 6. Pregnant woman diagnosed with ZIKV during this pregnancy using any criteria (clinical or laboratory diagnosis or self-report) as defined by the study 7. Any arbovirus-related symptoms during the current pregnancy 8. Birth weight in grams (<12 hours after delivery) 9. Abnormalities: neuro abnormalities, contractures, gastro abnormality, ocular, non-neurologic and congenital abnormality excluding microcephaly 10. Fetus confirmed diagnosis with ZIKV using any criteria (clinical or laboratory diagnosis or self-report) as defined by the study 11. Multiple vs single gestation 12. Current arbovirus infection 13. Prior arbovirus infection 14. History of preterm labor in any prior pregnancy 15. Diagnosis of congenital Zika syndrome (as measured by the study) at pregnancy or at birth 16. Miscarriage, loss, or live birth 17. Documented miscarriage: spontaneous loss of the product of the gestation <20 weeks 18. Microcephaly in fetus 19. Level of microcephaly (diagnosed in prenatal, infant or child period) as defined by the study. | Considering our a priori expectations, we anticipated that, for ZIKV-negative mothers, approximately 2.28% of newborns would have primary microcephaly, based on the “2 standard deviations below average” definition.^21^ To reflect this belief, we defined a Gaussian prior centered around the value of 2.28% as the most likely baseline prevalence of microcephaly in the overall population and we assign a 50% a priori probability to the baseline prevalence in the overall population falling within the range of 1.17% to 4.37%. The prevalence of microcephaly in non-infected populations is known to vary across different populations due to demographic differences and variations in the measurement of head circumference and gestational age (for further insights, refer to Silva et al.^30^ and Harville et al.^31^). Therefore, for each study-specific population, we established Gaussian priors that assign a 50% a priori probability for the baseline prevalence falling within the range of 0.53% to 9.30%.  For the fetal loss outcome, we defined a Gaussian prior corresponding to an *a priori* belief that about 2% is the most likely value for the risk of fetal loss in the overall population, and a 50% *a priori* probability that the risk in the overall population is within the range 1% to 3%, as described in the literature.^32,33,34^    For the CZS outcome, we defined a Gaussian prior corresponding to an *a priori* belief that 9% is the most likely value for the risk of CZS in the overall population (amongst women with ZIKV infection) and a 50% *a priori* probability that the risk in the overall population (amongst women with ZIKV infection) is within the range of 6% to 14%. This matches as closely as possible the 5 to 14% range cited in the literature.^35^    We incorporated weakly informative Gaussian priors for variables representing the association between the outcome (e.g., primary microcephaly, fetal loss) and maternal ZIKV infection at the overall population level. These priors were chosen to reflect an *a priori* belief that there is a 50% probability for the odds ratio, which measures the association, to fall within the range of 0.2 to 4.5. Given the substantial potential for variation in the association between the outcome and maternal ZIKV infection across study-specific populations, we defined Gaussian priors to capture the *a priori* belief that the odds ratio measuring the association in each study-specific population lies within the range of 0.1 to 8.9. |
| **Meta-analysis approach** | |
| We used both one-stage and two-stage IPD-MA approaches to estimate the ARs and RRs for primary and secondary outcomes.  In the two-stage meta-analysis, ARs and Wilson 95% confidence intervals were estimated per imputed dataset and per study, with continuity corrections applied separately for ZIKV-positive and ZIKV-negative pregnant individuals. We logit-transformed the risks and intervals and pooled across studies using Rubin’s rules, followed by back-transformation to the original scale.^22^ We pooled the results using a random-effects meta-analysis^23^, applying the Hartung-Knapp correction to the pooled estimates^24^ and presented the findings in forest plots. Confidence intervals for individual studies, as shown in the figures, are derived from the 2-stage meta-analysis.    We estimated RRs for studies that included both ZIKV-positive and ZIKV-negative women, only if at least one outcome occurred in both groups.    For the one-stage meta-analysis approach, we estimated mixed binomial models with a log link, incorporating ZIKV exposure as an independent variable and including a random intercept by study. | We used a Bayesian random-effects logistic regression model to analyze the data. This model establishes a relationship between the probability of an outcome (e.g., microcephaly) and maternal ZIKV infection through a logit link function^24^. The baseline probability of the outcome and the association between the exposure (i.e., maternal ZIKV infection) and the outcome may vary across different study populations. To account for this variation, we incorporated a hierarchical prior specification in the model employing weakly informative Gaussian priors for the regression coefficient parameters. These priors were selected to align with prior beliefs derived from the existing literature as closely as possible. For the variance parameters in the model, we adopted half-Normal priors following the recommendations of Gelman et al.^29^ We also defined priors to recognize the association between the ZIKV status using both the standardized and the ZIKV status given according to the study-specific definition. *A* *priori*, we believe sensitivity and specificity of the study-specific indicator as a surrogate for the standardized indicator to be relatively high and assign a 50% *a priori* probability that these falls within [0.83, 0.99]. |
| **Software used** | |
| We conducted all frequentist analyses using RStudio version 4.2.2 and R version 4.0.4 in a Linux environment. The 'mice', 'micemd', and 'miceadds' packages were used for the imputation process, and the 'EpiStats', 'metafor', and 'lme4' packages were employed for the meta-analysis.^25,26,27,28^ | We fit Bayesian models using JAGS (Just Another Gibbs Sampler), with three independent chains, each with ten thousand draws (20% burn-in and thinning of 10).^36^ |

# Table S5: Maternal ZIKV infection status and agreement between study definition and standardized definition

| **Cohort^§^** | **ZIKV study definition (N, %)**^¥^ | | | **ZIKV standardized definition (N, %)** ^¥^ | | | | | **Agreement^£^ between the 2 definitions** |
| --- | --- | --- | --- | --- | --- | --- | --- | --- | --- |
|  | **Negative** | **Positive** | **Unclassified** | **Negative** | **Limited** | **Moderate** | **Robust** | **Unclassified** |  |
| **003-GUF**  **(n=284)** | 0  (0) | 284  (100) | 0 (0) | 11 (3.9) | 0 (0) | 256 (91.8) | 12 (4.3) | 5 (1.8) | 🔵🔵🔵 0.89 |
| **004-ESP**  **(n=266)** | 202 (89.8) | 23  (10.2) | 41 (15.4) | 202 (89.8) | 0 (0) | 18 (8.0) | (2.2) | 41 (15.4) | 🔵🔵🔵0.98 |
| **005-ESP**  **(n=160)** | 118 (96.7) | 4  (3.3) | 38 (23.8) | 144 (90.0) | 0 (0) | 15 (9.4) | 1 (0.6) | 0 (0) | 🔵🔵⚪ 0.54 |
| **006-COL**  **(n=170)** | 0  (0) | 170  (100) | 0 (0) | 0 (0) | 0 (0) | 0 (0) | 170 (100) | 0 (0) | 🔵🔵🔵 1.00 |
| **007-COL**  **(n=56)** | 0  (0) | 52  (100) | 4 (7.1) | 0 (0) | 0 (0) | 20 (38.5) | 32 (61.5) | 4 (7.1) | 🔵🔵🔵 1.00 |
| **008-USA^×^**  **(n=56)** | 10  (22.7) | 34  (77.3) | 12 (21.4) | 10 (22.7) | 0 (0) | 23 (52.3) | 11 (25.0) | 12 (21.4) | 🔵🔵🔵 1.00 |
| **009-GRD**  **(n=242)** | 218 (90.1) | 24  (9.9) | 0 (0) | 18 (78.3) | 0 (0) | 0 (0) | 5 (21.7) | 219 (90.5) | ⚪⚪⚪ -0.81 |
| **010-BRA**  **(n=490)** | 0  (0) | 490  (100) | 0 (0) | 0 (0) | 0 (0) | 0 (0) | 490 (100) | 0 (0) | 🔵🔵🔵 1.00 |
| **011-BRA ^×^**  **(n=30)** | 6  (66.7) | 3  (33.3) | 21 (70.0) | 0 (0) | 0 (0) | 0 (0) | 1 (100) | 29 (96.7) | 🔵⚪⚪ 0.47 |
| **012-TTO**  **(n=66)** | 0  (0) | 66  (100) | 0 (0) | 2 (3.0) | 0 (0) | 0 (0) | 64 (97.0) | 0 (0) | 🔵🔵🔵 0.94 |
| **013-BRA ^×^**  **(n=653)** | 596 (91.3) | 57  (8.7) | 0 (0) | 0 (0) | 0 (0) | 3 (5.3) | 54 (94.7) | 596 (91.3) | ⚪⚪⚪ - 0.83 |
| **014-BRA**  **(n=46)** | 33  (71.7) | 13  (28.3) | 0 (0) | 0 (0) | 0 (0) | 0 (0) | 0 (0) | 46 (100) | ⚪⚪⚪ -1.00 |
| **015-BRA**  **(n=146)** | 41 (28.1) | 105 (71.9) | 0 (0) | 0 (0) | 0 (0) | 8 (100) | 0 (0) | 138 (94.5) | ⚪⚪⚪ -0.89 |
| **016-HND**  **(n=626)** | 334 (94.4) | 20  (5.7) | 272 (43.5) | 0 (0) | 0 (0) | 0 (0) | 6 (100) | 620 (99.0) | ⚪⚪⚪ -0.11 |
| **017-USA**  **(n=607)** | 0  (0) | 607  (100) | 0 (0) | 6 (1.0) | 0 (0) | 560 (93.7) | 32 (5.4) | 9 (1.5) | 🔵🔵🔵 0.95 |
| **018-COL ^×^**  **(n=1,180)** | 313 (55.4) | 252 (44.6) | 615 (52.1) | 194 (50.3) | 0 (0) | 48 (12.4) | 144 (37.3) | 794 (67.3) | 🔵🔵🔵 0.70 |
| **020-BRA**  **(n=55)** | 2  (3.6) | 53  (96.4) | 0 (0) | 9 (69.2) | 0 (0) | 0 (0) | 4 (30.8) | 42 (76.4) | ⚪⚪⚪ -0.78 |
| **021-PRI**  **(n=4,058)** | 0  (0) | 4058 (100) | 0 (0) | 8 (0.2) | 0 (0) | 2908 (71.7) | 1139 (28.1) | 3 (0.1) | 🔵🔵🔵 0.99 |
| **024-GTM**  **(n=381)** | 352 (93.6) | 24  (6.4) | 5 (1.3) | 347 (93.5) | 0 (0) | 24 (6.5) | 0 (0) | 10 (2.6) | 🔵🔵🔵 0.97 |
| **025-BRA**  **(n=54)** | 0 (0) | 54  (100) | 0 (0) | 0 (0) | 0 (0) | 0 (0) | 0 (0) | 54 (100) | ⚪⚪⚪ -1.00 |
| **026-KEN**  **(n=1,405)** | 1403 (99.9) | 2  (0.14) | 0 (0) | 1375 (99.4) | 0 (0) | 9 (0.7) | 0 (0) | 21 (1.5) | 🔵🔵🔵 0.96 |
| **027-BRA**  **(n=456)** | 114  (25) | 342  (75) | 0 (0) | 226 (60.1) | 0 (0) | 64 (17.0) | 86 (22.9) | 80 (17.5) | 🔵🔵🔵 0.65 ️ |

§ denominator = number of mothers; study sample sizes may differ from those in the original dataset, due to exclusions based on the criteria outlined in this manuscript.

¥ for headings with ZIKV results (Negative, Positive, Limited, Moderate, Robust, Unclassified): percentages are calculated excluding missing values from the denominator.

**×** indicates studies used for sensitivity analysis only

£ prevalence-Adjusted Bias-Adjusted Kappa: 🔵🔵🔵 (PAKAB > 0.6) strong agreement, 🔵🔵⚪ (PABAK 0.3–0.6) moderate agreement, 🔵⚪⚪ (PABAK 0–0.3) weak agreement, ⚪⚪⚪ (PABAK < 0) poor agreement.

# Table S6: Pregnancy and neonatal outcomes by study

| **Cohort**^§^ | **Women (N=9,568) / Children (n=9,608)** | **Microcephaly**^¥^ **- study definition (n, %)** | | | **CZS** ^¥^ **- study definition (n, %)** | | | **Fetal Loss** ^¥^ **- study definition (n, %)** | |
| --- | --- | --- | --- | --- | --- | --- | --- | --- | --- |
|  |  | **No** | **Yes** | **Missing** | **No** | **Yes** | **Missing** | **No** | **Yes** |
| **003-GUF** | 284/288 | 259 (90.0) | 29 (10.0) | 0 | 222 (90.2) | 24 (9.8) | 42 | 276 (97.9) | 6 (2.1) |
| **004-ESP** | 266/267 | 254 (96.2) | 10 (3.8) | 3 | 0 (0) | 0 (0) | 267 | 266 (100) | 0 (0) |
| **005-ESP** | 160/160 | 159 (99.4) | 1 (0.6) | 0 | 160 (100) | 0 (0) | 0 | 155 (100) | 0 (0) |
| **006-COL** | 170/170 | 141 (94.0) | 9 (6.0) | 20 | 147 (94.2) | 9 (5.8) | 14 | 156 (97.5) | 4 (2.5) |
| **007-COL** | 56/56 | 0 (0) | 39 (100) | 17 | 0 (0) | 0 (0) | 56 | 54 (96.4) | 2 (3.6) |
| **008-USA^×^** | 56/56 | 5 (10.2) | 44 (89.8) | 7 | 0 (0) | 0 (0) | 56 | 52 (92.9) | 4 (7.1) |
| **009-GRD** | 242/246 | 78 (31.8) | 167 (68.2) | 1 | 0 (0) | 7 (100) | 239 | 246 (100) | 0 (0) |
| **010-BRA** | 490/492 | 452 (92.4) | 37 (7.6) | 3 | 476 (97.3) | 13 (2.7) | 3 | 489 (100) | 0 (0) |
| **011-BRA ^×^** | 30/30 | 12 (40.0) | 18 (60.0) | 0 | 20 (69.0) | 9 (31.0) | 1 | 29 (96.7) | 1 (3.3) |
| **012-TTO** | 66/66 | 0 (0) | 0 (0) | 66 | 0 (0) | 0 (0) | 66 | 62 (96.9) | 2 (3.1) |
| **013-BRA ^×^** | 653/680 | 622 (96.1) | 25 (3.9) | 33 | 661 (99.7) | 2 (0.3) | 17 | 663 (97.9) | 14 (2.1) |
| **014-BRA** | 46/46 | 40 (87.0) | 6 (13) | 0 | 46 (100) | 0 (0) | 0 | 46 (100) | 0 (0) |
| **015-BRA** | 146/146 | 73 (83.9) | 14 (16.1) | 59 | 0 (0) | 0 (0) | 146 | 146 (100) | 0 (0) |
| **016-HND** | 626/630 | 573 (98.3) | 10 (1.7) | 47 | 599 (99.7) | 2 (0.3) | 29 | 592 (98.2) | 11 (1.8) |
| **017-USA** | 607/607 | 513 (91.8) | 46 (8.2) | 48 | 562 (97.7) | 13 (2.3) | 32 | 586 (100) | 0 (0) |
| **018-COL ^×^** | 1,180/1,192 | 1,145(96.1) | 47 (3.9) | 0 | 0 (0) | 0 (0) | 1,192 | 1,178 (99.3) | 8 (0.7) |
| **020-BRA** | 55/55 | 33 (61.1) | 21 (38.9) | 1 | 47 (87.0) | 7 (13.0) | 1 | 55 (100) | 0 (0) |
| **021-PRI** | 4,058/4,058 | 3,653 (92.9) | 282 (7.2) | 123 | 3,857 (98.2) | 71 (1.8) | 130 | 3,958 (100) | 0 (0) |
| **024-GTM** | 381/381 | 0 (0) | 0 (0) | 381 | 0 (0) | 0 (0) | 381 | 381 (100) | 0 (0) |
| **025-BRA** | 54/54 | 53 (100) | 0 (0) | 1 | 54 (100) | 0 (0) | 0 | 54 (100) | 0 (0) |
| **026-KEN** | 1,405/1,428 | 0 (0) | 0 (0) | 1,428 | 0 (0) | 0 (0) | 1,428 | 1,380 (97.5) | 35 (2.5) |
| **027-BRA** | 456/458 | 448 (97.8) | 10 (2.2) | 0 | 0 (0) | 0 (0) | 458 | 457 (100) | 0 (0) |

§ study sample sizes may differ from those in the original dataset, due to exclusions based on the criteria outlined in this manuscript. Country names are abbreviated using ISO 3166-1 alpha-3 codes.

¥ the denominator for fetal loss is derived from data concerning mothers with a gestational age of 20–42 weeks, while those for microcephaly is based on a gestational age of 24–42 weeks, consequently CZS is based on 24–42 weeks of gestation.

× indicates studies used for sensitivity analysis only

# Table S7: Newborns' birth statistics Based on the standardized ZIKV definition

| **Characteristic** | **ZIKV standardized definition (N, %)** | | | | |
| --- | --- | --- | --- | --- | --- |
|  | **Negative** | **Limited** | **Moderate** | **Robust** | **Unclassified** |
| **Newborns born to pregnant individuals with arbovirus-related symptoms** | 974/2,354  (41.4%) | 0  (0%) | 983/3,626  (27.1%) | 1,812/2,037  (25.4%) | 249/1,251  (19.9%) |
| **Newborns born to ZIKV-positive (study definition) pregnant individuals** | 168/2,337  (7.2%) | 0  (0%) | 3,867/3,88  (99.5%) | 2,048/2,050  (99.9%) | 328/977  (32.6%) |
| **Newborns from pregnant individuals with unknown ZIKV (study definition) status (N)** | 34 | 0 | 4 | 0 | 324 |

# Table S8: Sensitivity analysis with 23 studies: absolute and relative risk estimates based on different statistical analysis

| **Outcome** | **Estimate*** | **Frequentist** | | | |
| --- | --- | --- | --- | --- | --- |
|  |  | **ZIKV study definition (N, %)** | | **ZIKV standardized definition (N, %)** | |
|  |  | **One-stage** | **Two-stage** | **One-stage** | **Two-stage** |
| **Microcephaly at birth (2SD)** | **Absolute risk ZIKV+ (%)** | 5.0 [3.0,8.0] | 6.9 [4.0,11.5] | 4.2 [2.6,6.8] | 5.6 [3.2,9.5] |
|  | **Absolute risk ZIKV- (%)** | 2.2 [1.02,4.6] | 3.0 [1.3,6.6] | 4.4 [1.7,11.1] | 7.5 [2.9,18.2] |
|  | **Relative risk** | 1.5 [0.99,2.4] | 1.7 [1.04,2.7] | 0.7 [0.4,1.3] | 0.6 [0.3,1.2] |
| **Microcephaly at birth (3SD)** | **Absolute risk ZIKV+ (%)** | 1.7 [0.8,3.5] | 2.8 [1.4,5.6] | 1.3 [0.6,2.8] | 2.1 [1.02,4.4] |
|  | **Absolute risk ZIKV- (%)** | 0.3 [0.1,1.02] | 0.9 [0.2,3.0] | 0.7 [0.1,3.4] | 3.1 [0.7,12.8] |
|  | **Relative risk** | 3.9 [1.5,9.9] | 3.3 [1.3,8.7] | 0.98 [0.4,2.6] | 0.6 [0.2,1.8] |
| **Fetal Loss** | **Absolute risk ZIKV+ (%)** | 0.1 [0.01,1.3] | 2.4 [1.4,4.2] | 0.3 [0.1,1.3] | 2.2 [1.5,3.3] |
|  | **Absolute risk ZIKV-(%)** | 0.5 [0.1,2.0] | 2.2 [1.2,3.9] | 0.2 [0.01,6.15] | 2.9 [0.9,8.9] |
|  | **Relative risk** | 0.9 [0.4,2.0] | 1.1 [0.5,2.4] | 0.8 [0.3,2.7] | 0.6 [0.2,1.6] |
| **CZS (microcephaly 2SD)** | **Absolute risk ZIKV+ (%)** | 1.7 [0.8,3.7] | 3.0 [1.4,6.2] | 1.2 [0.5,2.7] | 2.3 [1.04,5.1] |
| **CZS (severe (3SD) microcephaly)** | **Absolute risk ZIKV+ (%)** | 1.04 [0.5,2.3] | 1.9[0.9,4.3] | 0.8 [0.3,1.8] | 1.7 [0.8,3.7] |
| **Congenital Zika** | **Absolute risk ZIKV+ (%)** | 12.1 [5.9,23.0] | 12.7 [7.6,20.6] | 10.1 [4.5,21.2] | 15.2 [6.7,31.0] |
