# Supplementary material for: Adverse fetal and perinatal outcomes associated with Zika virus infection during pregnancy: an individual participant data meta-analysis
Source: eClinicalMedicine. 2025 May 8;83:103231. doi: 10.1016/j.eclinm.2025.103231 (PMC12235389; doi:10.1016/j.eclinm.2025.103231)
Supplement: Collaborative authors [file mmc2.docx]

**Adverse fetal and perinatal outcomes associated with Zika virus infection during pregnancy: an individual participant data meta-analysis**

**The WHO Zika Virus Individual Participant Data Consortium: Authors list in alphabetical order**

| **FIRST NAME** | **LAST NAME** |
| --- | --- |
| Edna | Acosta Pérez |
| Juan | Aguilar Ticona |
| [Jackeline](mailto:karae@who.int) | Alger |
| Celia | Alpuche Aranda |
| Angélica | Amado Niño |
| [Emily](mailto:rabei@who.int) | Ansusinha |
| Thalia | Araújo |
| Juan | Arias |
| Lumumba | Arriaga Nieto |
| Marcos | Ávila |
| Azucena | Bardají |
| Carlos | Becerra Mojica |
| Andrea | Benedetti |
| Ana | Bertozzi |
| Sarah | Bethencourt |
| Karen | Blackmon |
| Victor | Borja Aburto |
| Patrícia | Brasil |
| Elizabeth | Brickley |
| William | Britt |
| Nathalie | Broutet |
| Pierre | Buekens |
| André | Cabié |
| David Alejandro | Cabrera-Gaytán |
| Rodrigo | Cachay |
| Maria Luisa | Cafferata |
| Isaac | Caicedo-Castro |
| Juan | Calle |
| Guilherme | Calvet |
| Harlan | Campbell |
| Maribel | Campos |
| Mabel | Carabali |
| Helen | Cerigo |
| Celia | Cordon |
| Juliana Silva | Corrêa |
| Federico | Costa |
| Conrado | Coutinho |
| Antonio | Cunha |
| Carlos | Cure Cure |
| Johanna | Damen |
| Marcela | Daza |
| Ilich | De la Hoz Siegler |
| Roberta | DeBiasi |
| Thomas | Debray |
| Valentijn | de Jong |
| Camille | Delgado-López |
| Geraldo | Duarte |
| Gabriella | Duarte Miranda |
| Valorie | Eckert |
| Sophie | Eickmann |
| Esther | Ellis |
| Cassia | Estofolete |
| Lester | Figueroa Bolaños |
| Olivier | Fléchelles |
| Kirsten | Fong |
| Maria Barbara | Franco Gomes |
| Trevon | Fuller |
| Victoria | Fumadó |
| Anna | Funk |
| Luis | Galvão |
| Gabriela Lopes | Gama |
| Patrick | Gérardin |
| Luz | Gibbons |
| Anna | Goncé |
| Amy | Gonzalez |
| Eduardo | Gotuzzo |
| Concepción | Grajales-Muñiz |
| Paul | Gustafson |
| Tahani | Hamdan |
| Eva | Harris |
| Cosme | Harrison |
| Najeh | Hcini |
| Cristina | Hofer |
| Natanaël | Holband |
| Claudia | Hormiga Sánchez |
| Ivonne | Huerta |
| Isabel | Hurtado |
| Irene | Inwani |
| David Cruvinel | Isaac |
| Thomas | Jaenisch |
| Esaú | João |
| Amadu | Juliana |
| Jose Paulo Pereira | Junior |
| Edna | Kara |
| Caron | Kim |
| Albert | Ko |
| Nancy | Krebs |
| Angelle Desiree | LaBeaud |
| Heather | Lake-Burger |
| Jill | Lebov |
| Yee-Sin | Leo |
| Brooke | Levis |
| Talita | Lima |
| Simon | Ling |
| Eduardo | Lopez-Medina |
| Cynthia | Lorenzo |
| Calum | Macpherson |
| Olivia | Manders |
| Elena | Marbán-Castro |
| Ernesto Torres de Azevedo | Marques |
| Celina Turchi | Martelli |
| Flor | Martinez-Espinosa |
| Gloria | Martinó González |
| Gustavo | Matta |
| Salim | Máttar |
| Lauren | Maxwell |
| John | McCracken |
| Adriana | Melo |
| Clara | Menéndez |
| Marcela | Mercado Reyes |
| María Consuelo | Miranda Montoya |
| Demócrito de Barros | Miranda-Filho |
| Ulisses | Montarroyos |
| Karel | Moons |
| Maria Elisabeth | Moreira |
| Jack | Moye |
| Sarah | Mulkey |
| José | Muñoz-Medina |
| [Johanna](mailto:isabeltrejos@westat.com) | Munoz |
| Marisa | Mussi-Pinhata |
| Silvia | Negrini |
| Nivison | Nery |
| Jurg | Niederbacher Velásquez |
| Karin | Nielsen |
| M. Kariuki | Njenga |
| Trevor | Noël |
| Mauricio | Nogueira |
| Theresa | Ochoa |
| Consuelo | Oliveira |
| Eric | Osoro |
| Ester | Paiva Souto |
| Miguel | Parra-Saavedra |
| Saulo | Passos |
| Luiza | Pela Rosado |
| Bernadete | Perez Coêlho |
| Priscila Cardia | Petra |
| Léo | Pomar |
| Arnaldo | Prata-Barbosa |
| Ingrid | Rabe |
| Mitermayer G. | Reis |
| Ana Maria | Rivera Casas |
| Diana | Rojas |
| Kerstin Daniela | Rosenberger |
| Paola Mariela | Saba Villarroel |
| Nuria | Sanchez Clemente |
| Magda | Sanz Cortes |
| Janet | Sayers |
| Deolinda | Scalabrin |
| Lavinia | Schuler-Faccini |
| Stacey | Schultz-Cherry |
| Aluisio | Segurado |
| Kirstin | Short |
| Priya | Shreedhar |
| Antônio | Silva |
| Ronaldo | Silva |
| Vivian Avelino | Silva |
| Isadora Cristina | de Siqueira |
| Karen | Sohan |
| Antoni | Soriano-Arandes |
| Carmen | Soria-Segarra |
| Paulo | Sucasas da Costa |
| Adriana | Tami |
| Jousilene | Tavares |
| Maria Benamor | Teixeira |
| Robert F. | Terry |
| Tun-Linn | Thein |
| Soe Soe | Thwin |
| Frank | Tobian |
| Vivian | Torres Rodríguez |
| Isabel | Trejos |
| Marilia Dalva | Turchi |
| César | Ugarte-Gil |
| Miguel | Valencia-Prado |
| Alfonso | Vallejos-Parás |
| Zilton | Vasconselos |
| Ana Beatriz Gorini | da Veiga |
| Carmen | Viñuela-Benéitez |
| Manon | Vouga |
| Yinghui | Wei |
| Jamie | Westcott |
| Marc-Alain | Widdowson |
| Ricardo | Ximenes |
| Carmen | Zorrilla |
